# Body size and symbiotic status influence gonad development in *Aiptasia pallida* anemones

**DOI:** 10.1007/s13199-016-0456-1

**Published:** 2016-10-29

**Authors:** Judith F. Carlisle, Grant K. Murphy, Alison M. Roark

**Affiliations:** Department of Biology, Furman University, 3300 Poinsett Highway, Greenville, SC 29613 USA

**Keywords:** Cnidaria, Dinoflagellate, Gonad, Oocyte, Sperm follicle, Zooxanthellae

## Abstract

Pale anemones (*Aiptasia pallida*) coexist with dinoflagellates (primarily *Symbiodinium minutum*) in a mutualistic relationship. The purpose of this study was to investigate the role of these symbionts in gonad development of anemone hosts. Symbiotic and aposymbiotic anemones were subjected to light cycles that induced gametogenesis. These anemones were then sampled weekly for nine weeks, and gonad development was analyzed histologically. Anemone size was measured as mean body column diameter, and oocytes or sperm follicles were counted for each anemone. Generalized linear models were used to evaluate the influence of body size and symbiotic status on whether gonads were present and on the number of oocytes or sperm follicles produced. Body size predicted whether gonads were present, with larger anemones being more likely than smaller anemones to develop gonads. Both body size and symbiotic status predicted gonad size, such that larger and symbiotic anemones produced more oocytes and sperm follicles than smaller and aposymbiotic anemones. Overall, only 22 % of aposymbiotic females produced oocytes, whereas 63 % of symbiotic females produced oocytes. Similarly, 6 % of aposymbiotic males produced sperm follicles, whereas 60 % of symbiotic males produced sperm follicles. Thus, while gonads were present in 62 % of symbiotic anemones, they were present in only 11 % of aposymbiotic anemones. These results indicate that dinoflagellate symbionts influence gonad development and thus sexual maturation in both female and male *Aiptasia pallida* anemones. This finding substantiates and expands our current understanding of the importance of symbionts in the development and physiology of cnidarian hosts.

**Electronic supplementary material:**

The online version of this article (doi:10.1007/s13199-016-0456-1) contains supplementary material, which is available to authorized users.

## Introduction

*Aiptasia pallida* (recently renamed *Exaiptasia pallida*; Grajales and Rodríguez 2016) is a species of anemone found along the Atlantic, Pacific, and Gulf coasts of North America and in the Caribbean Sea. Like many anthozoans, these anemones harbor intracellular dinoflagellates (commonly called zooxanthellae) of the genus *Symbiodinium* (primarily *S. minutum*; LaJeunesse et al. 2012; Thornhill et al. 2013). It is assumed that these photosynthetic symbionts primarily confer metabolic benefits to the host (Yellowlees et al. 2008), as a large proportion of the organic products of photosynthesis (primarily glycerol, along with simple sugars, amino acids, organic acids, glycoproteins, and possibly lipids) can be translocated to the anemone in exchange for inorganic nutrients (reviewed in Muller-Parker and Davy 2001; Davy et al. 2012).

In response to a variety of stressors including heat, cold, and irradiation, anthozoans undergo a process called bleaching in which the host expels its symbionts and becomes partially or wholly aposymbiotic (Steen and Muscatine 1987; Brown et al. 1995; Hobbs et al. 2013). In many cnidarians, this process is associated with slowed growth and diminished health (Hoegh-Guldberg 1999), and severe bleaching often leads to death of the host (Glynn 1983; Goreau et al. 2000; Hobbs et al. 2013). However, laboratory-reared *A. pallida* anemones can persist indefinitely in the aposymbiotic state, in part by increasing the number of cnidocytes and/or mucocytes as a specific mechanism for increasing prey capture efficiency after bleaching has occurred (Fransolet et al. 2013). Bleached anemones may thus be able to compensate, at least partially, for the lack of symbionts by increasing their reliance on heterotrophically acquired nutrients, as has been shown in corals (Tremblay et al. 2015).

Like many anemones, *A. pallida* reproduces both asexually and sexually (Hessinger and Hessinger 1981; Bocharova and Kozevich 2011). Asexual reproduction in *A. pallida* occurs through pedal laceration, a form of budding in which a portion of the pedal disk detaches from the parent anemone. This fragment includes portions of the parent’s mesenteries and lateral body wall and develops into a miniature, adult form within several days (Hessinger and Hessinger 1981; Bocharova and Kozevich 2011). Asexual reproduction facilitates rapid colonization of available habitats and provides a mechanism for replicating genotypes that have already proven successful in the present environment. On the other hand, the meiosis and recombination events of sexual reproduction promote the maintenance of genetic diversity within a species (Schlesinger et al. 2010). Sexual reproduction in many anthozoans occurs through broadcast spawning, in which eggs and sperm are released into the water where they fuse externally. In male anemones, spermatogenesis is often synchronous. Primary spermatocytes mature in the mesoglea, or acellular gelatinous layer between the epidermis and gastrodermis. As primary spermatocytes develop into secondary spermatocytes, spermatids, and then mature spermatozoa, these cells slowly migrate towards the middle of a sperm follicle, leaving less mature cells around the periphery of the follicle. Unlike spermatogenesis, oogenesis in anemones is often asynchronous. Primordial oogonia within the gastrodermis migrate into the mesoglea, differentiate into primary oocytes, and then mature into ova (Scott and Harrison 2009; Bocharova and Kozevich 2011).

Reproductive performance of cnidarian hosts is influenced by the presence of dinoflagellate symbionts. For example, the presence of symbionts may increase the rate of asexual reproduction of host anemones (Clayton 1985); however, their effects on sexual reproduction of host anemones are unknown. The purpose of this study was to test the hypothesis that gonad development and thus sexual function of the anemone host are influenced by the presence of dinoflagellate symbionts, as has been shown in corals (Szmant and Gassman 1990; Ward et al. 2002). Over the course of three gametogenic cycles, each lasting four weeks, both symbiotic and aposymbiotic anemones were evaluated weekly using histological techniques. Generalized linear models were then used to test whether anemone size and/or symbiotic status predicted whether gonads were present and the number of oocytes or sperm follicles produced. Our study provides important insight into the role of intracellular dinoflagellate symbionts in the development and physiology of their cnidarian hosts.

## Methods

*Aiptasia pallida* anemones with mixed genetic backgrounds were purchased from Carolina Biological Supply in the spring of 2013. These anemones were maintained as asexually propagating clone lines in 30 ppt Instant Ocean sea water in 70 × 50 mm Pyrex glass crystallizing dishes with glass lids. Anemones were fed brine shrimp (*Artemia spp.*) nauplii to satiation twice weekly and their dishes were cleaned two or three times each week. Aposymbiotic anemones were generated by cold-shocking symbiotic anemones in May of 2013 and then maintaining these asexually propagating clone lines in the dark while on the same feeding and cleaning schedule as described above for two years. At the start of the study and at the midpoint of the study, the aposymbiotic status of these anemones was confirmed using an EVOS FL imaging system (Advanced Microscopy Group, Mill Creek, Washington) with a custom light filter (excitation: 470 nm; emission: 685 nm) that causes algal cells to autofluoresce.

Beginning in May of 2015, dishes of both symbiotic and aposymbiotic anemones were maintained at 26.0 °C in an Intellus Control System incubator (Percival, Perry, IA). The normal light cycle consisted of fluorescent lighting (Philips Alto II F25T8/TL841 25-watt bulbs, color temperature of 4100 K, light output of 2150 lumens, 4 bulbs per 6256 cm^2^ shelf, mounted 21–23 cm above each shelf) on a 12 h:12 h light:dark cycle. For ten minutes immediately before and after each photophase, LED lunar lights (Current USA TrueLumen white lunar lights, color temperature of 12,000 K, six strips per 6256 cm^2^ shelf, mounted 21–23 cm above each shelf) were used to simulate dawn and dusk, respectively. Gametogenesis was induced by three consecutive 28-day cycles, each consisting of the normal light cycle for 23 consecutive days and a 16 h:8 h light:dark photoperiod with LED lunar lights on during the entire scotophase for five consecutive days (S. Perez, personal communication; Grawunder et al. 2015). The first day of the 16 h:8 h light:dark photoperiod was called Day 1 of each 28-day cycle.

Beginning two weeks after Day 1 of the first 28-day cycle, asexually propagated anemones (total *n* = 69) from 27 clone lines of known sex were sampled in each of nine consecutive weeks. Of these anemones, nine were aposymbiotic females from four clone lines, 18 were aposymbiotic males from seven clone lines, 27 were symbiotic females from eight clone lines, and 15 were symbiotic males from eight clone lines. Gonad development was then evaluated histologically. Anemones were first anesthetized in a 1:1 solution of 0.37 M MgCl_2_ and 30 ppt seawater at ambient temperature. Each anemone was then immersed in 10 % seawater-buffered formalin (Fisher Scientific, Pittsburgh, PA). After 24 hours, each anemone was transferred to a Fisherbrand® cassette. Fixed anemones in cassettes were then rinsed in deionized water and serially dehydrated in a two-day process (Online Resource 1). Tissue-Tek® stainless steel base molds were coated with glycerin jelly, and anemones were embedded in these molds in paraffin wax with their tentacles facing upwards. Paraffin-embedded anemones were then sectioned using an American Optical 820 Spencer Microtome (Rankin Biomedical Corporation, Holly, MI) at a thickness of 7 μm and mounted onto Fisher Superfrost Plus microscope slides. Odd-numbered slides were subjected to hematoxylin-eosin staining (Online Resource 2), and even-numbered slides of usable sections were stained with a modified Masson trichrome stain (Online Resource 3). Stained sections were analyzed under a light microscope. A photograph of the single image with the largest number of oocytes or sperm follicles for each anemone was taken with a Canon EOS Rebel Xsi camera (Canon U.S.A., Inc., Melville, NY). Images were analyzed using ImageJ (Abramoff et al. 2004). The presence or absence of oocytes or sperm follicles was scored as binary data, and the number of oocytes or sperm follicles was counted for each anemone. Anemone size was quantified as the mean of three measurements of body column diameter.

Data for female and male anemones were analyzed separately using R v3.2.2 (R Core Team 2014; code available in Online Resource 4). Because no differences in gonad development were observed across weeks, data were not analyzed separately by week. Body column diameters were tested for normality using Shapiro tests, and variances were compared using Bartlett tests. Because the assumptions of normality and equality of variance were not satisfied, the diameters of aposymbiotic and symbiotic anemones were compared using Mann–Whitney U tests. A Spearman correlation test was used to evaluate the relationship between the number of oocytes or sperm follicles and body column diameter for symbiotic females or symbiotic males, respectively. Binary data (presence or absence of oocytes or sperm follicles) and count data (number of oocytes or sperm follicles) were analyzed with generalized linear models using binomial and Poisson distributions, respectively. Models were compared using Akaike’s information criterion (AIC) values and multimodel inference (Burnham and Anderson 2002). Potential model covariates included individual and additive terms of anemone size and symbiotic status. AIC values were generated for each model. Models were ranked and compared by ∆AIC to determine the model(s) (∆AIC < 2) that best predicted our binomial and count data. When two top models were identified, these models were averaged (full average) using the MuMIn package in R.

## Results

**Fig. 1:**
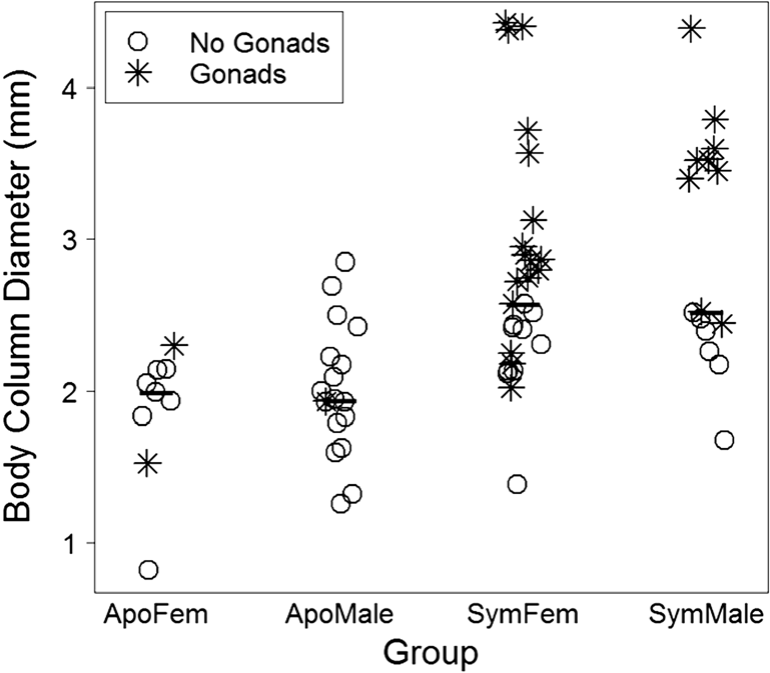
Sizes of *Aiptasia pallida* anemones used in this study, quantified as means of three measurements of the body column of each anemone. *Bold dashes* represent medians, *open circles* represent anemones without gonads, and *asterisks* represent anemones with gonads. Body column diameter was larger in symbiotic anemones than in aposymbiotic anemones for both males and females (Mann–Whitney U tests, *p* < 0.001). Sample size: *n* = 9 aposymbiotic females, *n* = 18 aposymbiotic males, *n* = 27 symbiotic females, and *n* = 15 symbiotic males. Abbreviations: ApoFem = aposymbiotic female, ApoMale = aposymbiotic male, SymFem = symbiotic female, SymMale = symbiotic male

**Fig. 2:**
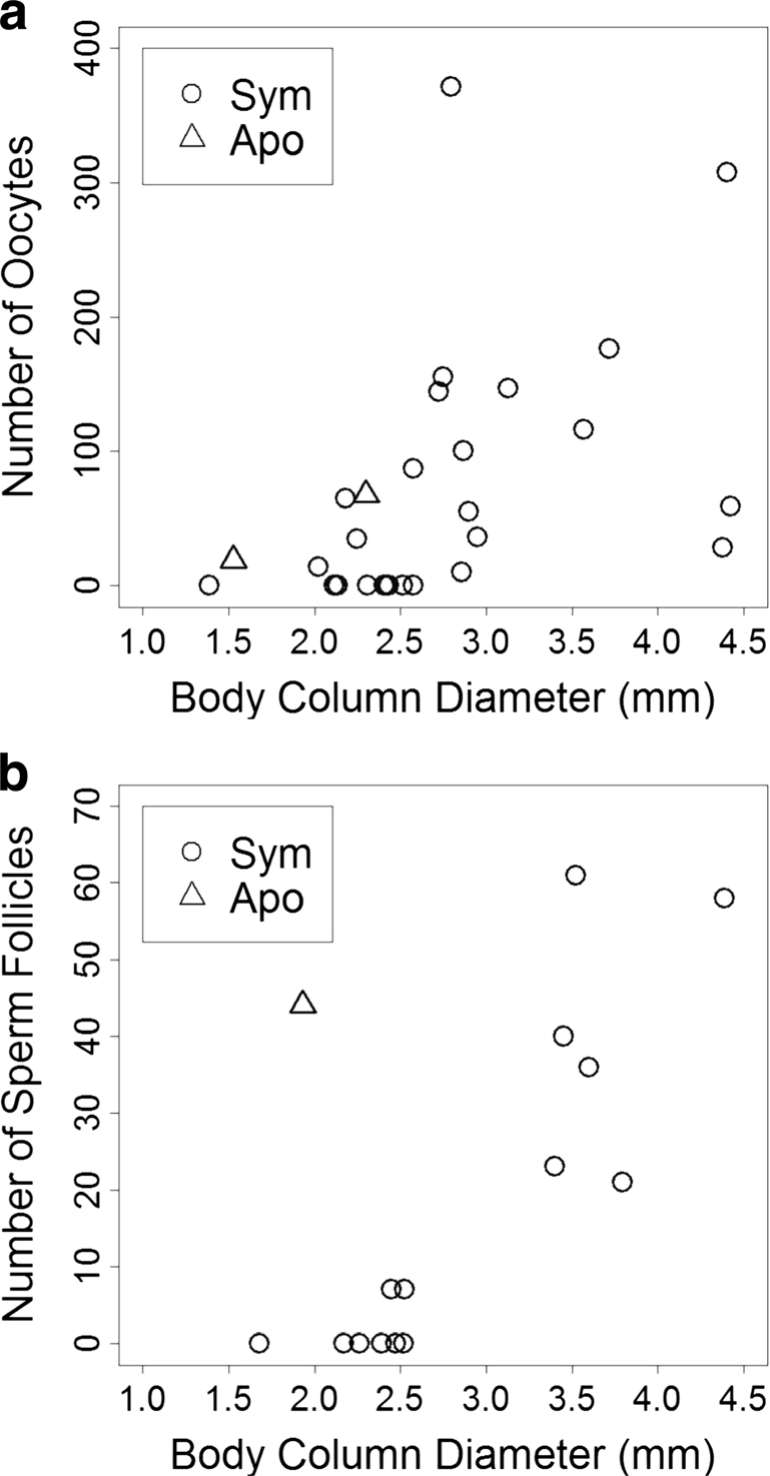
**a**) Number of oocytes and body column diameter in mature female anemones (*n* = 27 symbiotic anemones, 1 aposymbiotic anemone) and **b**) number of sperm follicles and body column diameter in mature male anemones (*n* = 15 symbiotic anemones, 2 aposymbiotic anemones). Number of oocytes and number of sperm follicles were both positively correlated with body column diameter in symbiotic anemones (Spearman correlation test; oocytes: *ρ* = 0.654, *p* < 0.001; sperm follicles: *ρ* = 0.833, *p* < 0.001). SYM = symbiotic, APO = aposymbiotic

Body column diameter was larger for symbiotic (median = 2.58 mm) than for aposymbiotic (median = 1.99 mm) female anemones (Mann–Whitney U test, *p* < 0.0001; Fig. 1). Body column diameter was also larger for symbiotic (median = 2.53 mm) than for aposymbiotic (median = 1.94 mm) male anemones (Mann–Whitney U test, *p* < 0.001; Fig. 1). In symbiotic females, the number of oocytes was positively correlated (*ρ* = 0.654, *p* < 0.001; Fig. 2a) with body column diameter. In symbiotic males, the number of sperm follicles was positively correlated (*ρ* = 0.833, *p* < 0.001; Fig. 2b) with body column diameter. Correlation tests could not be run on aposymbiotic females or males because only two aposymbiotic females had oocytes and only one aposymbiotic male had sperm follicles.

**Fig. 3:**
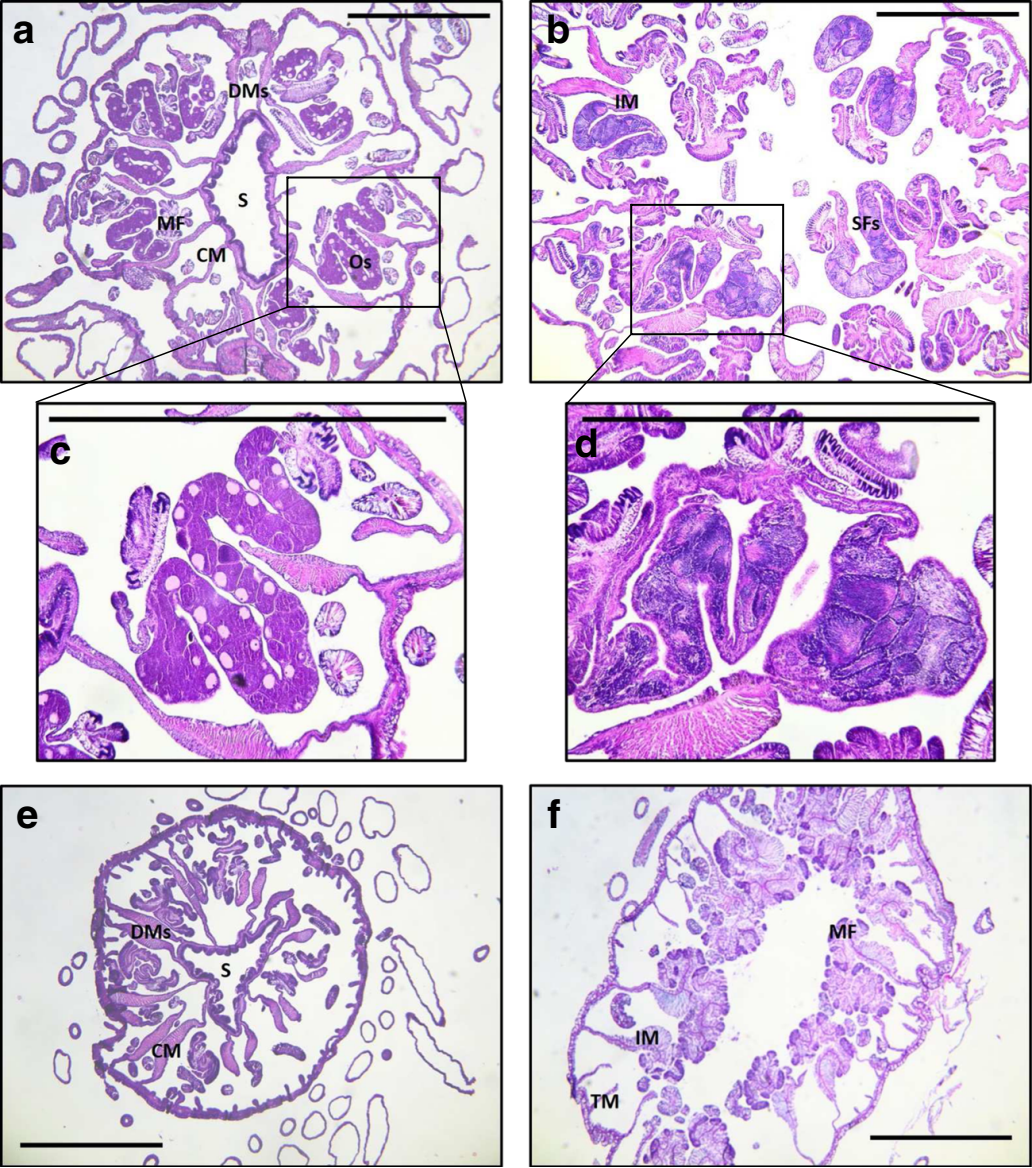
Representative histological sections of sampled anemones. **a**) Female symbiotic anemone with oocytes present; **b**) male symbiotic anemone with sperm follicles present; **c**) magnified inset of panel a; **d**) magnified inset of panel b; **e**) female aposymbiotic anemone with no oocytes present; **f**) male aposymbiotic anemone with no sperm follicles present. Anemones were sectioned at 7 μm and stained with hematoxylin and eosin (see Online Resource 2). Scale bar = 1 mm. Abbreviations: CM = complete mesentery, DMs = directive mesenteries, IM = incomplete mesentery, MF = mesenterial filament, Os = oocytes, S = siphonoglyph, SFs = sperm follicles, TM = tertiary mesentery

**Fig. 4:**
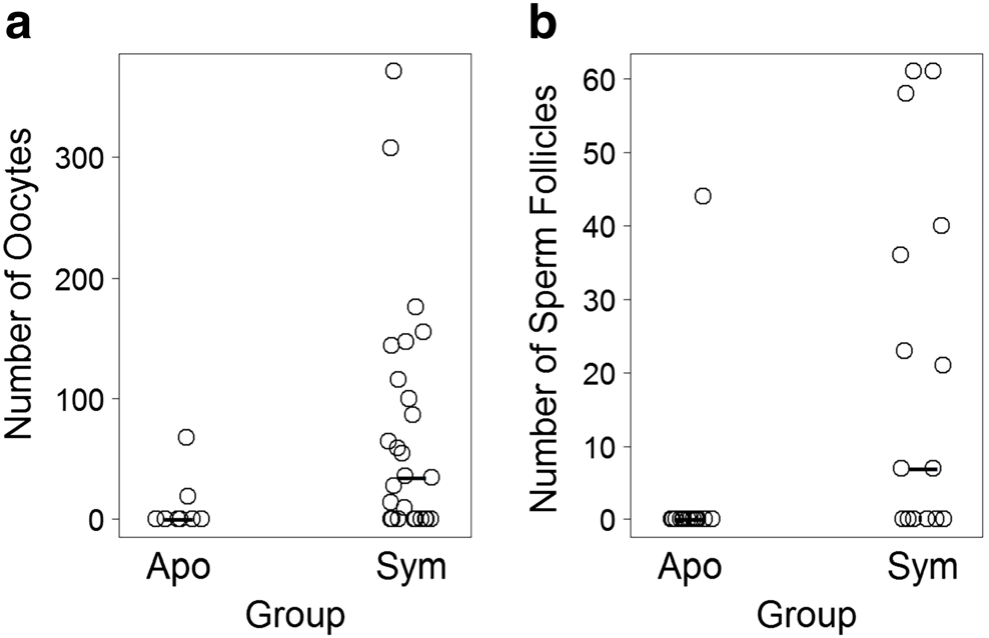
**a**) Number of oocytes in females and **b**) number of sperm follicles in males. Each *open circle* represents one anemone. *Bold dashes* represent medians. Sample size: *n* = 9 aposymbiotic females, *n* = 27 symbiotic females, *n* = 18 aposymbiotic males, and *n* = 15 symbiotic males. Abbreviations: Apo = aposymbiotic, Sym = symbiotic

Body column diameter predicted whether gonads were present in females (Table 1) and males (Table 2). Specifically, larger anemones were more likely than smaller anemones to have either oocytes or sperm follicles present. Both body column diameter and symbiotic status predicted the number of oocytes in females (Table 1) and the number of sperm follicles in males (Table 2). Specifically, larger and symbiotic anemones produced more oocytes and sperm follicles than smaller and aposymbiotic anemones (Figs. 3 and 4). Only 22 % (2 of 9) of aposymbiotic females produced oocytes, whereas 63 % (17 of 27) of symbiotic females produced oocytes (Fig. 4a). Similarly, 5.6 % (1 of 18) of aposymbiotic males produced sperm follicles, whereas 60 % (9 of 15) of symbiotic males produced sperm follicles (Fig. 4b). Overall, while gonads were present in 61.9 % of symbiotic anemones, they were present in only 11.1 % of aposymbiotic anemones.

**Table 1:** (A) AIC model selection results and (B) parameter estimates generated from generalized linear models used to predict whether oocytes were present (based on a binomial distribution) and the number of oocytes present (based on a Poisson distribution) in female anemones. Model selection results are presented in order of increasing AIC values, with the top models (∆AIC < 2) indicated with an asterisk (*). Parameter estimates with standard errors for the top models are shown, with non-zero estimates (based on 95% confidence intervals) indicated with a bullet (•). Abbreviations: k = number of parameters, AIC = Akaike’s Information Criterion, ∆AIC = difference in AIC value from model #1 to each model, Sym = symbiotic

A. Model selection results				B. Model Parameters		
Binomial Model	*k*	AIC	ΔAIC	Averaged Top Models	Estimate	Std. Error
1. *Size	2	37.6	0.0	Intercept	-7.631•	2.935
2. *Size + Group	3	39.5	1.9	Size	3.188•	1.262
3. Group	2	49.1	11.5	Group (Sym)	-0.036	0.560
4. Null	1	51.8	14.2			
Poisson Model	*k*	AIC	ΔAIC	Top Model	Estimate	Std. Error
1. *Size + Group	3	2782.9	0.0	Intercept	0.907•	0.119
2. Size	2	2932.4	149.5	Size	0.711•	0.026
3. Group	2	3463.6	680.7	Group (Sym)	1.218•	0.115
4. Null	1	4084.4	1301.5

**Table 2:** (A) AIC model selection results and (B) parameter estimates generated from generalized linear models used to predict whether sperm follicles were present (based on a binomial distribution) and the number of follicles present (based on a Poisson distribution) in male anemones. Model selection results are presented in order of increasing AIC values, with the top models (∆AIC < 2) indicated with an asterisk (*). Parameter estimates with standard errors for the top models are shown, with non-zero estimates (based on 95% confidence intervals) indicated with a bullet (•). Abbreviations: k = number of parameters, AIC = Akaike’s Information Criterion, ∆AIC = difference in AIC value from model #1 to current model, Sym = symbiotic

A. Model selection results				B. Model Parameters		
Binomial Model	*k*	AIC	ΔAIC	Averaged Top Models	Estimate	Std. Error
1. *Size	2	23.7	0.0	Intercept	-9.853•	3.367
2. *Size + Group	3	24.1	0.4	Size	3.362•	1.322
3. Group	2	31.9	8.2	Group (Sym)	0.629	1.140
4. Null	1	42.5	18.8			
Poisson Model	*k*	AIC	ΔAIC	Top Model	Estimate	Std. Error
1. *Size + Group	3	488.9	0.00	Intercept	-1.822•	0.241
2. Size	2	498.3	9.40	Size	1.281•	0.084
3. Group	2	754.0	265.1	Group (Sym)	0.653•	0.198
4. Null	1	1033.7	544.8

## Discussion

Intracellular dinoflagellate symbionts provide photosynthetically derived nutrients and thus metabolic support to their cnidarian hosts (Bergschneider and Muller-Parker 2008). These nutrients presumably facilitate host sexual function, as gonad development is constrained by symbiont loss in corals (Szmant and Gassman 1990; Michalek-Wagner and Willis 2001a, 2001b; Bingham et al. 2014). However, the effects of symbiont loss on reproductive function in actiniarians have not been well studied. The purpose of this laboratory study was to determine whether the presence of symbionts influences gonad development and thus sexual reproductive capacity of *Aiptasia pallida* anemones. We found that symbiotic status affected both body size and gonad development, as symbiotic anemones tended to be larger with more extensive gonads compared to aposymbiotic anemones.

In our study, the presence or absence of gonads was primarily determined by body size, with larger anemones being more likely to develop gonads than smaller anemones. Not surprisingly, aposymbiotic anemones were generally smaller than symbiotic anemones, although the size ranges of both groups overlapped. Symbionts translocate to their cnidarian hosts a substantial proportion of photosynthetically fixed, high energy organic compounds (Muller-Parker and Davy 2001; Davy et al. 2012). In *A. pallida*, the loss of these symbionts is associated with decreased expression of many genes, including those for carbohydrate, lipid, and amino acid transporters (Lehnert et al. 2014). Thus, the detrimental effects of symbiont loss on reproductive capacity that we identified appear to result, at least in part, from decreased nutrient acquisition. As the rate of photosynthesis positively correlates with the rate of heterotrophy in *A. pallida* hosting naturally occurring *Symbiodinium minutum* symbionts (Leal et al. 2015), aposymbiotic anemones may also have eaten less and thus been doubly disadvantaged by symbiont loss. On the other hand, aposymbiotic anemones may have compensated for the loss of symbionts by increasing their reliance on heterotrophically acquired nutrients, as has been demonstrated in corals (Grottoli et al. 2006; Tremblay et al. 2015). Relative rates of heterotrophy could not be compared in our study because we did not quantify intake or nutrient retention.

Severe reductions in symbiont populations are associated with decreased host size in other anemone species (Hobbs et al. 2013) and with decreased lipid and protein content of host tissues in corals. The decreased lipid and protein contents of coral polyps with reduced symbiont populations are magnified in their eggs (Michalek-Wagner and Willis 2001b), which are smaller and less nutrient-dense as well as developmentally delayed compared to eggs of polyps with normal symbiont populations (Michalek-Wagner and Willis 2001a). These results indicate that nutrient limitation of adults can have profound implications for the production and survival of offspring, a finding that is substantiated by our data. Although we did not quantify host body composition, the loss of photosymbionts was associated with decreased anemone size, and smaller anemones were less likely to develop gonads than larger anemones. Thus, the induction of gametogenesis in aposymbiotic *A. pallida* is likely constrained by nutrient limitation, as has been suggested for both soft corals (Michalek-Wagner and Willis 2001b) and scleractinian corals (Szmant and Gassman 1990).

Body size was a substantial predictor of whether gonads were present in our anemones, but symbionts did not influence reproductive capacity of their hosts solely through effects on body size. Instead, gonad size, which was quantified as the number of sperm follicles and oocytes present in males and females, respectively, was determined by a combination of both body size and symbiotic status. Consequently, large and symbiotic anemones had larger gonads than small and aposymbiotic anemones. A similar pattern was observed in female *Hydra viridis*, in which oogenesis was induced only in polyps containing symbiotic zoochlorellae. Interestingly, and contrary to our results, male aposymbiotic *H. viridis* polyps produce spermaries, indicating that symbionts influence gametogenesis in female but not in male hydra (Habetha et al. 2003).

Although symbiotic status was identified as a predictor of gonad size in our study, the high AIC values associated with the Poisson predictive models indicate poor model fit, particularly for females. These high AIC values were likely driven by the very small number of aposymbiotic anemones that developed gonads. Overall, only two aposymbiotic female anemones produced oocytes and one aposymbiotic male anemone produced sperm follicles. Incidentally, the two female aposymbiotic anemones that produced oocytes were from the same clone line; the aposymbiotic status of this particular clone line could not be confirmed by fluorescent microscopy prior to sampling. Thus, these two females (and perhaps the lone aposymbiotic male with sperm follicles) may have in fact harbored some symbionts that facilitated gonad development.

This study provides additional evidence that gonad development in host cnidarians is influenced by symbiotic status; it also provides further insight into the sublethal effects of diminished symbiont populations in these animals. Our finding that the effects of symbiotic status do not result from differences in body size alone is particularly novel and suggests that symbiotic anemones, which acquire nutrients through both host heterotrophy and symbiont autotrophy, are able to allocate more nutrients to reproduction than aposymbiotic anemones of the same size. Future studies should explore the relationship between heterotrophy and autotrophy and their effects on body composition and gonad development in symbiotic cnidarians.

## Electronic supplementary material

Below is the link to the electronic supplementary material.

ESM 1 (PDF 89 kb)

ESM 2 (PDF 92 kb)

ESM 3 (PDF 167 kb)

ESM 4 (PDF 14.8 kb)
