# Supplementary material for: Body size and symbiotic status influence gonad development in Aiptasia pallida anemones
Source: Symbiosis. 2016 Oct 29;71(2):121–7. doi: 10.1007/s13199-016-0456-1 (PMC5277023; doi:10.1007/s13199-016-0456-1)
Supplement: Supplementary file 1 — (PDF 89 kb) [file 13199_2016_456_MOESM1_ESM.pdf]

Body size and symbiotic status influence gonad development in *Aiptasia pallida* anemones
Symbiosis
Judith F. Carlisle, Grant K. Murphy, Alison M. Roark
Corresponding author: Alison M. Roark, 864-294-3757, alison.roark@furman.edu
Furman University, Department of Biology, 3300 Poinsett Highway, Greenville, SC 29613 USA

Protocol used to serially dehydrate anemone tissue after fixation but prior to embedding, including the solution, duration, and relevant brand information

Solution	Duration	Brand Used
1. Deionized water	15 min.	
2. Running tap water	4 hr.	
3. 50% ethanol	3 hr.	Pharmco Aaper (Belmont, NC)
4. 70% ethanol	3 hr. or overnight	Pharmco Aaper
5. 85% ethanol	45 min.	Pharmco Aaper
6. 95% ethanol I	45 min.	Pharmco Aaper
7. 95% ethanol II	45 min.	Pharmco Aaper
8. 100% ethanol	45 min.	Pharmco Aaper
9. 100% ethanol	45 min.	Pharmco Aaper
10. 100% ethanol	45 min.	Pharmco Aaper
11. Citrisolv I	45 min.	Fisher Scientific (Pittsburgh, PA)
12. Citrisolv II	45 min.	Fisher Scientific
13. Histoplast paraffin wax I	2 hr. (vacuum at 10 in. Hg at 62°C)	Fisher Scientific
14. Histoplast paraffin wax II	2 hr. (vacuum at 5 in. Hg at 62°C)	Fisher Scientific
