# Supplementary material for: Body size and symbiotic status influence gonad development in Aiptasia pallida anemones
Source: Symbiosis. 2016 Oct 29;71(2):121–7. doi: 10.1007/s13199-016-0456-1 (PMC5277023; doi:10.1007/s13199-016-0456-1)
Supplement: Supplementary file 2 — (PDF 92 kb) [file 13199_2016_456_MOESM2_ESM.pdf]

Body size and symbiotic status influence gonad development in *Aiptasia pallida* anemones

Symbiosis

Judith F. Carlisle, Grant K. Murphy, Alison M. Roark
Corresponding author: Alison M. Roark, 864-294-3757, alison.roark@furman.edu
Furman University, Department of Biology, 3300 Poinsett Highway, Greenville, SC 29613 USA

Protocol for hematoxylin-eosin staining after sectioning and mounting, including the solution, duration, and relevant brand information

Solution	Duration	Brand Used
1. CitriSolv I	2 min.	Fisher Scientific (Pittsburgh, PA)
2. CitriSolv II	2 min.	Fisher Scientific
3. CitriSolv III	2 min.	Fisher Scientific
4. 100% ethanol	10 dips	Pharmco Aaper (Belmont, NC)
5. 95% ethanol	10 dips	Pharmco Aaper
6. 70% ethanol	10 dips	Pharmco Aaper
7. Running water	3 min.	
8. Harris hematoxylin	2 min.	Fisher Scientific
9. Running tap water	3 min.	
10. Scott solution	2 min.	Fisher Scientific
11. Running water	3 min.	
12. Eosin Y	1 min.	Sigma Aldrich (St. Louis, MO)
13. 70% ethanol	1 dip	Pharmco Aaper
14. 95% ethanol	3 dips	Pharmco Aaper
15. 100% ethanol	2 min.	Pharmco Aaper
16. 100% ethanol	2 min.	Pharmco Aaper
17. CitriSolv I	2 min.	Fisher Scientific
18. CitriSolv II	2 min.	Fisher Scientific
19. CitriSolv III	Until coverslipped	Fisher Scientific
