# Supplementary material for: Body size and symbiotic status influence gonad development in Aiptasia pallida anemones
Source: Symbiosis. 2016 Oct 29;71(2):121–7. doi: 10.1007/s13199-016-0456-1 (PMC5277023; doi:10.1007/s13199-016-0456-1)
Supplement: Supplementary file 3 — (PDF 167 kb) [file 13199_2016_456_MOESM3_ESM.pdf]

Body size and symbiotic status influence gonad development in *Aiptasia pallida* anemones
Symbiosis
Judith F. Carlisle, Grant K. Murphy, Alison M. Roark
Corresponding author: Alison M. Roark, 864-294-3757, alison.roark@furman.edu
Furman University, Department of Biology, 3300 Poinsett Highway, Greenville, SC 29613 USA

Protocol for modified Masson trichrome staining after sectioning and mounting, including the solution, duration, and relevant brand information

Solution	Duration	Brand Used
1. Citrisolv I	3 min.	Fisher Scientific (Pittsburgh, PA)
2. Citrisolv II	3 min.	Fisher Scientific
3. Citrisolv III	3 min.	Fisher Scientific
4. 100% ethanol	3 min.	Pharmco Aaper (Belmont, NC)
5. 100% ethanol	3 min.	Pharmco Aaper
6. 100% ethanol	3 min.	Pharmco Aaper
7. 95% ethanol	3 min.	Pharmco Aaper
8. 80% ethanol	3 min.	Pharmco Aaper
9. Deionized water	5 min.	
10. Bouin's solution	Overnight in hood	ScyTek Laboratories Inc. (Logan, UT)
11. Wash (running water) and rinse for 1 min (deionized water)	Wash until yellow disappears	
12. Weigert's iron hematoxylin	5 min.	Sigma Aldrich (St. Louis, MO)
13. Wash (running water) and rinse for 1 min (deionized water)	5 min.	
14. Biebrich scarlet-acid fuchsin	5 min.	ScyTek Laboratories Inc.
15. Deionized water	1 min.	
16. Phosphomolybdic/phosphotungstic acid	10 min.	ScyTek Laboratories Inc.
17. Aniline blue	5 min.	ScyTek Laboratories Inc.
18. Deionized water	1 min.	
19. 1% acetic acid	3-5 min.	Ricca Chemical (Arlington, TX)
20. 95% ethanol I	3 min.	Pharmco Aaper
21. 95% ethanol II	3 min.	Pharmco Aaper
22. 100% ethanol I	3 min.	Pharmco Aaper
23. 100% ethanol II	3 min.	Pharmco Aaper
24. Citrisolv I	5 min.	Fisher Scientific
25. Citrisolv II	5 min.	Fisher Scientific
26. Citrisolv III	5 min.	Fisher Scientific
27. Permount (for coverslipping)	Let sit overnight to dry	Fisher Scientific
