# Supplementary material for: Body size and symbiotic status influence gonad development in Aiptasia pallida anemones
Source: Symbiosis. 2016 Oct 29;71(2):121–7. doi: 10.1007/s13199-016-0456-1 (PMC5277023; doi:10.1007/s13199-016-0456-1)
Supplement: Supplementary file 4 — (PDF 14.8 kb) [file 13199_2016_456_MOESM4_ESM.pdf]

Body size and symbiotic status influence gonad development in *Aiptasia pallida* anemones
Symbiosis
Judith F. Carlisle, Grant K. Murphy, Alison M. Roark
Corresponding author: Alison M. Roark, 864-294-3757, alison.roark@furman.edu
Furman University, Department of Biology, 3300 Poinsett Highway, Greenville, SC 29613 USA

Relevant R code:

```
#MALE SIZE
males<-read.csv("boxplot males.csv")
by(males$MeanSize, males$Group, shapiro.test)
bartlett.test(MeanSize~Group, data=males)
wilcox.test(MeanSize~Group, data=males)
par(mai=c(1.2,1.5,0.5,4))
stripchart(males$Follicles~males$Group, vertical=TRUE, method="jitter", pch=1, cex=2, xlab="",
        ylab="", las=1, cex.lab=1.6, cex.axis=1.5)
points(c(1,2), tapply(males$Follicles, males$Group, median), pch="__", cex=4)
mtext("Number of Sperm Follicles",side=2,line=3.5, cex=2, at=30)
mtext("Group", side=1, line=3, cex = 2)
par(mai=c(1.4,1.6,0.5,0.5))
plot(FolliclesSym~MeanSize, data=males, pch=1, cex=2, xlab="", ylab="", cex.axis=2.5, mgp=c(3,1.5,0),
        ylim=c(0,70), xlim=c(1,4.5))
lines(FolliclesApo~MeanSize, data=males, type="p",pch=2, cex=2, xlab="", ylab="", cex.axis=2.5,
        mgp=c(3,1.5,0), ylim=c(0,70), xlim=c(1,4.5))
leg.txt<-c("Sym", "Apo")
legend(1, 70, leg.txt,pch=c(1,2), pt.cex=2, cex=1.5)
mtext("Number of Sperm Follicles",side=2,line=3.9, cex=3, at=35)
mtext("Body Column Diameter (mm)", side=1, line=4, cex = 3, at=2.75)
cor.test(males$FolliclesSym, males$MeanSize, method="spearman")

#FEMALE SIZE
females<-read.csv("boxplot females.csv")
by(females$MeanSize, females$Group, shapiro.test)
bartlett.test(MeanSize~Group, data=females)
wilcox.test(MeanSize~Group, data=females)
par(mai=c(1.2,1.5,0.5,4))
stripchart(females$Oocytes~females$Group, vertical=TRUE, method="jitter", pch=1, cex=2, xlab="",
        ylab="", las=1, cex.lab=1.6, cex.axis=1.5)
points(c(1,2), tapply(females$Oocytes, females$Group, median), pch="__", cex=4)
mtext("Number of Oocytes",side=2,line=3.5, cex=2, at=195)
mtext("Group", side=1, line=3, cex = 2)
par(mai=c(1.4,1.6,0.5,0.5))
plot(OocytesSym~MeanSize, data=females, pch=1, cex=2, xlab="", ylab="", cex.axis=2.5,
        mgp=c(3,1.5,0), ylim=c(0,400), xlim=c(1,4.5))
lines(OocytesApo~MeanSize, data=females, type="p", pch=2, cex=2, xlab="", ylab="", cex.axis=2.5,
        mgp=c(3,1.5,0), ylim=c(0,400), xlim=c(1,4.5))
leg.txt<-c("Sym", "Apo")
legend(1, 400, leg.txt,pch=c(1,2), pt.cex=2, cex=1.5)
mtext("Number of Oocytes",side=2,line=3.9, cex=3, at=200)
mtext("Body Column Diameter (mm)", side=1, line=4, cex = 3, at=2.75)
cor.test(females$OocytesSym, females$MeanSize, method="spearman")#
```

```
STRIP CHART WITH SEPARATE PLOTS
alldata<-read.csv("anemone data one column.csv")
par(mai=c(1.2,1.2,0.5,0.5))
stripchart(alldata$MeanSize~alldata$Group, vertical=TRUE, method="jitter",
	pch="",xlab="Group",ylab="Body Column Diameter (mm)", las=1, cex.lab=1.6, cex.axis=1.5)
points(c(1,2,3,4), tapply(alldata$MeanSize, alldata$Group, median), pch="__", cex=4)
stripchart(alldata$MeanSize[alldata$YesNo=="no"] ~ alldata$Group[alldata$YesNo=="no"],
	method="jitter", vertical=TRUE, pch=1, col ="black", cex=2, las=1, add=TRUE)
stripchart(alldata$MeanSize[alldata$YesNo=="yes"] ~ alldata$Group[alldata$YesNo=="yes"],
	method="jitter", vertical=TRUE, pch=8, col="black", cex=2, las=1, add=TRUE)
leg.txt<-c("No Gonads", "Gonads")
legend(0.72, 4.5, leg.txt, pch=c(1,8), pt.cex=2, cex=1.5)

# GENERALIZED LINEAR MODELS
# install package ("MuMIn")
library(MuMIn)

# FEMALES
females<-read.csv("boxplot females.csv")
gmfemale1<-glm(Oocytes~MeanSize+Group, females, family="poisson")
gmfemale2<-glm(Oocytes~MeanSize, females, family="poisson")
gmfemale3<-glm(Oocytes~Group, females, family="poisson")
gmfemale4<-glm(Oocytes~1, females, family="poisson")
AIC(gmfemale1,gmfemale2,gmfemale3,gmfemale4)
summary(gmfemale1)
gmpfemale1<-glm(OocytesBinomial~MeanSize+Group, females, family="binomial")
gmpfemale2<-glm(OocytesBinomial~MeanSize, females, family="binomial")
gmpfemale3<-glm(OocytesBinomial~Group, females, family="binomial")
gmpfemale4<-glm(OocytesBinomial~1, females, family="binomial")
AIC(gmpfemale1,gmpfemale2,gmpfemale3,gmpfemale4)
averagegmpfemale<-model.avg(gmpfemale2,gmpfemale1,revised.var=TRUE)
summary(averagegmpfemale)

# MALES
males<-read.csv("boxplot males.csv")
gmmale1<-glm(Follicles~MeanSize+Group, males, family="poisson")
gmmale2<-glm(Follicles~MeanSize, males, family="poisson")
gmmale3<-glm(Follicles~Group, males, family="poisson")
gmmale4<-glm(Follicles~1, males, family="poisson")
AIC(gmmale1,gmmale2,gmmale3,gmmale4)
summary(gmmale1)
gmpmale1<-glm(FolliclesBinomial~MeanSize+Group, males, family="binomial")
gmpmale2<-glm(FolliclesBinomial~MeanSize, males, family="binomial")
gmpmale3<-glm(FolliclesBinomial~Group, males, family="binomial")
gmpmale4<-glm(FolliclesBinomial~1, males, family="binomial")
AIC(gmpmale1,gmpmale2,gmpmale3,gmpmale4)
averagegmpmale<-model.avg(gmpmale2,gmpmale1,revised.var=TRUE)
summary(averagegmpmale)
```
